# The Australian Diagnostic Criteria for Contrast-Induced Encephalopathy

**DOI:** 10.1007/s00234-025-03601-5

**Published:** 2025-03-29

**Authors:** Frederick P. Mariajoseph, Leon T. Lai, Adrian Praeger, Ronil V. Chandra, Justin Moore, Hamed Asadi, Laetitia de Villiers, Tony Goldschlager, Calvin Gan, Kevin Zhou, Albert Ho Yuen Chiu, Ferdinand Miteff, Ramon Martin Banez, Thanh Phan, Davor Pavlin-Premrl, Winston Chong, Sophie Dunkerton, Anoop Madan, Lee-Anne Slater

**Affiliations:** 1 Monash University, Melbourne , Australia; 2 Monash Health, Melbourne, Australia; 3 Austin Health, Melbourne, Australia; 4 Vincent’S Hospital, Melbourne, Australia; 5 Gold Coast Hospital, Southport, Australia; 6 Sir Charles Gairdner Hospital, Perth, Australia; 7 University of Western Australia, Perth, Australia; 8 Hunter New England Local Health District, Newcastle, Australia; 9 Royal Hobart Hospital, Hobart, Australia; 10 Alfred Health, Melbourne, Australia; 11 Royal Brisbane and Women’S Hospital, Brisbane, Australia

**Keywords:** Contrast, Neurotoxicity, Encephalopathy, Complication, Adverse event, Endovascular, Diagnosis

## Abstract

**Introduction:**

Contrast-induced encephalopathy (CIE) is a recognised complication of contrast administration, however diagnosis remains challenging due to its symptom overlap with other neurological conditions and the absence of formal diagnostic criteria.

**Methods:**

A modified Delphi study was performed. Consultant physicians with active clinical experience with CIE patients were invited from neurovascular centres in Australia. Initial diagnostic items were derived from an extensive literature review and analysis of local institutional cases across Australia. Three Delphi rounds were conducted. Consensus was defined as ≥ 75% agreement.

**Results:**

Seventeen neurovascular specialists from nine neurovascular centres participated (81.0% response rate) between May 2024 and July 2024. In round 1, 15 diagnostic items were presented to participants, which were revised and one additional criteria suggested. In round 2, 14/16 diagnostic items achieved consensus. In round three 14/14 items achieved consensus. Ultimately, a 14-item diagnostic criteria was developed based on participant consensus. The absolute criteria exclude CIE if symptom onset is more than 24 h after contrast administration, or if symptoms can be explained by vessel occlusion/territory ischaemia, intracranial haemorrhage, epilepsy, metabolic derangement, intracranial malignancy or head trauma. The supporting criteria indicate that CIE is more probable if symptoms are reversible, correspond with the distribution of contrast administration, or are associated with reversible contrast staining, cerebral oedema or cortical/subcortical MRI signal change.

**Conclusion:**

This study proposes a 14-item diagnostic criteria for CIE based on expert consensus in Australia. Further research is needed to refine CIE as a clinical entity.

**Supplementary Information:**

The online version contains supplementary material available at 10.1007/s00234-025-03601-5.

## Introduction

With the increasing use of contrast in diagnostic imaging and endovascular interventions, contrast-induced encephalopathy (CIE) has become a more frequently observed complication of iodinated contrast administration. CIE, also known as contrast-induced neurotoxicity (CIN), presents as a spectrum of neurological symptoms including cortical blindness, aphasia, focal motor deficits, seizures, and reduced consciousness, often resembling stroke [1–8]. CIE is a rare, but established complication of endovascular procedures, with one systematic review estimating its incidence to be 0.51% (95% CI 0.3%—1.0%)[4], although the incidence appears to be higher following endovascular neurointervention [9]. Diagnosing CIE is challenging, with a recent survey indicating that less than a third of clinicians feel confident in making the diagnosis [10]. The lack of a standardized diagnostic criteria further exacerbates this difficulty. To address this, we conducted a modified Delphi study to develop diagnostic criteria for CIE, in order to enhance clinical decision-making.

## Methods

### Study design

This study was approved by the Monash Health ethical review board (RES-24–0000-111Q). Suitable participants were invited from neurovascular centres across Australia. All participants provided voluntary and express consent to be included in this study. Panel members were selected based on clinical experience with CIE patients. Physicians in neurovascular centres across Australia were invited and assess for their experience with CIE in clinical practice. Eligibility criteria required participants to be (i) consultant/attending physicians in a relevant specialty, (ii) currently in active clinical practice, and (iii) have direct clinical experience in diagnosing and managing CIE patients.

### Literature review

A broad literature review was conducted of Medline (1946 to June 2023), PubMed (1946 to June 2023), and Embase (1947 to June 2023). Key search terms included “contrast”, “neurotoxicity”, “encephalopathy”, “blindness” and “deficit”. Search terms were combined utilising Boolean operators as appropriate. Cases of CIE in the literature were examined alongside over 50 identified cases in Australia. Factors including onset of symptoms, clinical features and radiological findings were reviewed to develop the initial diagnostic items. The literature review was conducted by the survey moderator (FPM), and the panel members were blinded to the development of the initial diagnostic items (supplementary Table 1).

### Modified delphi study design

This study was conducted according to the proposed methodologic criteria for Delphi studies [11]. A three-round online survey was conducted from May 2024 to July 2024. The survey was distributed using *google forms*, and responses were collated in *google sheets* (Google LLC, Mountain View, California, USA). In the first round, participants were presented with a list of diagnostic items derived from the literature review and the identified local cases, with Diagnostic criteria categorized as “absolute” (essential for CIE diagnosis) and “supporting” (indicative but not required for CIE diagnosis). Participants were asked to rate their agreement based on a five-point Likert scale. Participants were asked to provide reasons for items that they disagreed with. Moreover, participants were asked to provide comments about modifying existing criteria and to suggest additional diagnostic items not included in the original list. Demographic data were also collected in Round 1.

In the second round, diagnostic items were revised based on participants' comments and suggestions from the first round, including newly suggested criteria. Participants rated their agreement with the revised items using a five-point Likert scale. Items that reached consensus were advanced to the third round, while those that did not were excluded. Aggregate data of each round was presented to participants in the subsequent round. Following the third round, participants were asked to specify the number of supporting criteria required for CIE to be classified as a probable diagnosis. All responses were anonymous and participants were blinded to other’s individual responses.

### Statistical analysis

All statistical calculations were conducted by the survey moderator (FPM). Agreement was calculated as the percentage of positive responses (i.e. agree or strongly agree) when compared to all responses. Consensus was defined a priori as 75% or more of participants rating an item as agree or strongly agree. Following each survey round, the moderator calculated agreement for each diagnostic item, and presented it in the subsequent survey round. Items that did not reach consensus (i.e. < 75% agreement) were not carried forward. All statistical analyses were performed with Stata/BE (StataCorp LLC, College Station, Texas, USA).

## Results

Seventeen physicians participated in this study (81.0% response rate), all of whom were in active clinical practice with experience treating CIE patients (Table 1). Specialties included interventional neuroradiology (64.7%), neurosurgery (17.6%) and neurology (17.6%). All 17 participants completed the three survey rounds.

**Table 1:** Participant characteristics

Variable	n (%)
Current clinical practice	17 (100)
Active experience with CIE patients	17 (100)
Specialty	
Interventional Neuroradiology	11 (64.7)
Neurosurgery	3 (17.6)
Neurology	3 (17.6)
Years of independent practice	
< 5 years	4 (23.5)
5–10 years	4 (23.5)
10–20 years	6 (35.3)
> 20 years	3 (17.6)

The participants were initially presented with 15 diagnostic items. In Round 1, the participants suggested adding one additional diagnostic item, as well as suggested the rewording of some of the initial diagnostic items. Following Round 1, a total of 16 diagnostic items were identified. In Round 2, two diagnostic items did not reach consensus amongst participants, and hence were excluded. The remaining 14 items reached consensus in Round 3 (Table 2), of which 5 diagnostic items were supporting criteria. The median number of criteria required to make CIE a probable diagnosis was 3.These items were combined to form the diagnostic criteria, which was approved by all participants.

**Table 2:** Results of Delphi Rounds 2 and 3

Diagnostic Item	Agreement (%)		Included (Y/N)
	*Round 2*	*Round 3*	
CIE should only be diagnosed if presentation is within 24 h of contrast administration	100	100	Y
CIE should not be diagnosed if neurological signs can be explained by the presence of vessel occlusion or vascular territory ischaemia	88	94	Y
Punctate diffusion restriction not correlating to symptoms does not necessitate vascular territory ischaemia	100	94	Y
Perfusion abnormality without large vessel occlusion does not necessitate vascular territory ischaemia	82	100	Y
CIE should not be diagnosed if neurological signs can be explained by intracranial haemorrhage	94	100	Y
CIE should not be diagnosed if neurological signs s can be explained by a pre-existing seizure pattern in patients with known epilepsy	82	88	Y
CIE should not be diagnosed if neurological signs can be explained by metabolic derangement	94	94	Y
CIE should not be diagnosed if neurological signs can be explained by the presence of intracranial malignancy	94	94	Y
CIE should not be diagnosed if neurological signs can be explained by infection or delirium	64	N/A	N
CIE should not be diagnosed if neurological signs can be explained by recent head trauma	76	82	Y
CIE is more likely if neurological symptoms are reversible	100	100	Y
CIE is more likely if neurological signs correlate to the vascular territory/anatomical distribution that was exposed to contrast	100	100	Y
CIE is more likely if neuroimaging is normal	58	N/A	N
CIE is more likely in the presence of contrast staining that resolves on subsequent imaging	94	94	Y
CIE is more likely in the presence of cerebral oedema resolves on subsequent imaging	88	94	Y
CIE is more likely in the presence of cortical and subcortical signal MRI signal changes that resolve on subsequent imaging	88	94	Y

The finalised diagnostic criteria is presented in Table 3. The absolute criteria exclude CIE if symptom onset is more than 24 h after contrast administration, or if symptoms can be attributed to vessel occlusion/territory ischaemia, intracranial haemorrhage, epilepsy, metabolic derangement, intracranial malignancy or head trauma. The supporting criteria suggest that CIE is more likely if symptoms are reversible, correlate with the distribution of contrast administration or are associated with reversible imaging findings including contrast staining, cerebral oedema or cortical/subcortical MRI signal change.

**Table 3:** The Australian Diagnostic Criteria for Contrast-Induced Neurotoxicity

Absolute Criteria:
1. Presentation is < 24 h after contrast administration
2. Neurological signs cannot be explained by the presence of vessel occlusion or vascular territory ischaemia, noting:
a. Punctate diffusion restriction on diffusion-weighted MRI, not correlating to symptoms, does not necessarily represent significant vascular territory ischaemia for the purpose of this exclusionary criteria, clinical discretion is advised
b. Perfusion abnormality on CT or MR perfusion, without vessel occlusion, does not necessarily represent significant vascular territory ischaemia for the purpose of this exclusionary criteria, clinical discretion is advised
3. Neurological signs cannot be explained by intracranial haemorrhage
4. Neurological signs cannot be explained by a pre-existing seizure pattern in patients with known epilepsy
5. Neurological signs cannot be explained by biochemical derangement
6. Neurological signs cannot be explained by the presence of intracranial malignancy
7. Neurological signs cannot be explained by recent head trauma
**Supporting Criteria:**
1. Neurological signs are reversible
2. Neurological signs correlate to the vascular territory of direct contrast injection (in neurointervention)
3. There is contrast staining, that subsequently resolves
4. There is cerebral oedema, that subsequently resolves
5. There is cortical or subcortical MRI signal changes, that subsequently resolves
***Diagnosis***
• ***Possible CIE* = *All absolute criteria are met*** • ***Probable CIE *= *All absolute criteria are met AND* ≥ *3 supporting criteria are met***

## Discussion

Despite increasing reports of CIE in the literature and in clinical practice, standardized protocols for diagnosis are lacking. Using a modified Delphi study with Australian neurovascular clinicians, we developed a diagnostic criteria to provide a uniform framework for enhancing diagnostic accuracy and consistency in clinical practice.

CIE is challenging to diagnose due to its symptom overlap with other neurological conditions. The proposed criteria emphasize the need to differentiate CIE from other potentially significant and time-urgent pathologies. The absolute criteria are stringent. CIE is excluded if symptoms appear more than 24 h after contrast administration as delayed onset is not considered clinically consistent in the opinion of the authors. CIE is also excluded if symptoms can be clearly attributed to other neurological pathologies including ischaemic stroke, intracranial haemorrhage, epilepsy, biochemical derangement (e.g. hypoglycaemia, hyponatraemia), malignancy and head trauma. These exclusions prevent incorrect CIE diagnosis in conditions that require prompt treatment.

**Fig. 1:**
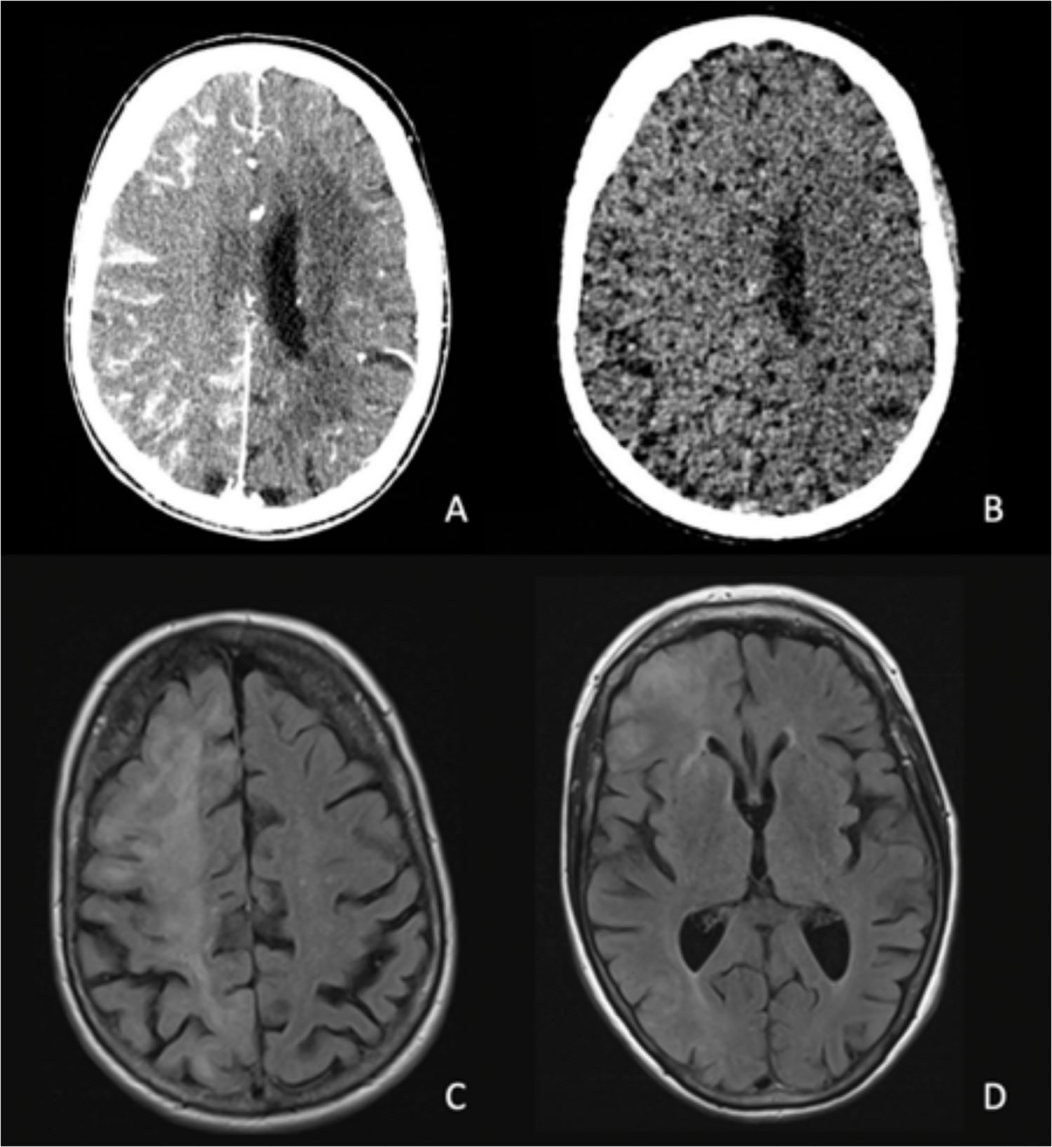
Hemispheric contrast staining and sulcal effacement/cerebral oedema (**A**, **B**), subcortical and cortical FLAIR signal change (concordant diffusion restriction must be excluded) (**C**, **D**)

The supporting criteria focus on both the clinical and radiological aspects of CIE. Contrast staining and cerebral oedema, as well as MR signal change represent the most common radiological findings of CIE [12] (Fig. 1). MR signal change has been observed across a several sequences and anatomical locations, but appears to most commonly be described as FLAIR hyperintensity in cortical and subcortical regions [12]. It must also be noted that a large proportion of patients do have normal neuroimaging, and hence an absence of abnormalities on CT or MR do not preclude a diagnosis of CIE. The supporting criteria ultimately serve to highlight the dynamic nature of CIE, noting that symptoms are more likely to be explained by CIE when they are reversible and accompanied by transient radiological findings such as contrast staining and cerebral oedema. The diagnostic terminology reflects the need for this delayed, repeat clinical and radiological assessment to come to a higher level of confidence in the diagnosis of CIE.

The pathophysiology of CIE, may be described in two key phases. Firstly, the blood–brain barrier (BBB) is disrupted, allowing passage of contrast agents into the central nervous system (CNS), which is usually protected [13, 14]. This is subsequently followed by neurotoxic effects of contrast agents, by disruption of the electrophysiology and metabolism of the CNS [15]. Damage to the BBB may be secondary to clinical factors such as hypertension or prior ischaemic stroke, as well as secondary to contrast agents themselves [16–21]. Hypertension leads to increased cerebrovascular shear stress, which may affect the integrity of the BBB, with its association with CIE has been demonstrated in clinical cohorts [5, 17]. Similarly, ischaemic stroke is known to damage, likely due to hypoxic damage of tight junctions and the vascular endothelium [21, 22]. Further investigation to improve the understanding of the pathophysiology of CIE and its risk factors, will provide significant insight.

The presentation of CIE is variable, but is stereotypically characterised by symptom onset within hours, followed by resolution of symptoms within days. The literature describes the common manifestations of CIE to include cortical blindness, confusion, hemiparesis, aphasia, and seizures [12]. Median time of symptom onset has been reported as 1 h post-procedurally, with a recent systematic review describing complete resolution of symptoms in 85% of patients, and the majority achieving complete symptom resolution within 72 h of symptom onset [23]. Once diagnosed, the management of CIE is largely empirical, with no standardized treatment protocols due to limited understanding of its pathophysiology and variability in the literature. Common treatments include intravenous fluids [24, 25] to expedite contrast removal from the cerebrovasculature and corticosteroids to protect the BBB [23, 26]. Supportive treatments may include mannitol for cerebral oedema [24, 27] and anticonvulsants for seizures [28, 29].

This study has several limitations. Firstly, although the Delphi method is a validated study design, it is inherently subjective, and therefore the consensus achieved may not necessarily reflect the most accurate or comprehensive approach to diagnosis. Secondly, the sample size of 17 participants, although sufficient for the Delphi method, may not fully capture the diversity of experience within the broader clinical community. Additionally, the panel was exclusively composed of Australian neurovascular clinicians, which may limit the generalisability of the proposed criteria to other regions with different clinical practices. Finally, excluding criteria that did not reach consensus might omit relevant diagnostic factors with clinical significance. Further investigation is needed to validate the proposed criteria in diverse settings and larger cohorts to enhance its robustness and applicability. Future studies should focus on validating the criteria through retrospective and prospective investigation, by applying it to cohorts of patients who have been clinically diagnosed with CIE. In addition, the diagnostic accuracy of the criteria may be assessed by examining interobserver reliability in distinguishing CIE from other neurological entities.

## Conclusion

This study proposes a 14-item diagnostic criteria for contrast-induced encephalopathy (CIE), based on the consensus of Australian neurovascular specialists with clinical experience in CIE. Further investigation is needed to validate these criteria and deepen our understanding of CIE as a clinical entity.

## Supplementary Information

Below is the link to the electronic supplementary material.

Supplementary file1 (DOCX 13 KB)

## Data Availability

No datasets were generated or analysed during the current study.

## References

[1] Aykan A, Zehir R, Karabay CY, Kocabay G (2012) Contrast− induced monoplegia following coronary angioplasty with iopromide. Kardiologia Polska (Polish Heart J) 70(5):499–500 22623245

[2] Deb-Chatterji M, Schafer L, Grzyska U, Gelderblom M (2018) Stroke-mimics: An acute brainstem syndrome after intravenous contrast medium application as a rare cause of contrast-induced neurotoxicity. Clin Neurol Neurosurg 174:244–246 30308472 10.1016/j.clineuro.2018.10.002

[3] Quintas-Neves M, Araújo JM, Xavier SA, Amorim JM, Cruz E Silva V, Pinho J (2020) Contrast-induced neurotoxicity related to neurological endovascular procedures: a systematic review. Acta Neurologica Belgica 120 419-24 10.1007/s13760-020-01508-x 32997325

[4] Vazquez S, Graifman G, Spirollari E, Ng C, Uddin A, Feldstein E et al (2022) Incidence and risk factors for acute transient contrast-induced neurologic deficit: a systematic review with meta-analysis. Stroke Vascular Interventional Neurol 2(1):e000142 10.1161/SVIN.121.000142 PMC12778740 41585285

[5] Zevallos CB, Dandapat S, Ansari S, Farooqui M, Quispe-Orozco D, Mendez-Ruiz A et al (2020) Clinical and imaging features of contrast-induced neurotoxicity after neurointerventional surgery. World Neurosurgery 142:e316–e324 32634632 10.1016/j.wneu.2020.06.218

[6] Niimi Y, Kupersmith M, Ahmad S, Song J, Berenstein A (2008) Cortical blindness, transient and otherwise, associated with detachable coil embolization of intracranial aneurysms. Am J Neuroradiol 29(3):603–607 18065506 10.3174/ajnr.A0858 PMC8118896

[7] Allison C, Sharma V, Park J, Schirmer CM, Zand R (2021) Contrast-induced encephalopathy after cerebral angiogram: a case series and review of literature. Case Reports in Neurol 13(2):405–413 10.1159/000516062 PMC8255655 34248578

[8] Dattani A, Au L, Tay KH, Davey P (2018) Contrast-induced encephalopathy following coronary angiography with no radiological features: a case report and literature review. Cardiology (Switzerland) 139(3):197–201 10.1159/000486636 29402812

[9] Mariajoseph FP, Lai LT, Moore J, Chandra RV, Goldschlager T, Praeger A et al (2024) Incidence of contrast-induced neurotoxicity following endovascular treatment of unruptured intracranial aneurysms: a single-centre cohort study. Acta Neurol Belg 124(6):1989–1994 39325269 10.1007/s13760-024-02643-5 PMC11614943

[10] Mariajoseph FP, Lai LT, Moore J, Chandra RV, Goldschlager T, Praeger A et al (2023) Current knowledge and perspectives of contrast-induced neurotoxicity: A survey of Australian clinicians. J Clin Neurosci 116:8–12 37597332 10.1016/j.jocn.2023.08.014

[11] Diamond IR, Grant RC, Feldman BM, Pencharz PB, Ling SC, Moore AM et al (2014) Defining consensus: a systematic review recommends methodologic criteria for reporting of Delphi studies. J Clin Epidemiol 67(4):401–409 24581294 10.1016/j.jclinepi.2013.12.002

[12] Mariajoseph FP, Yu D, Lai LT, Moore J, Goldschlager T, Chandra RV et al (2024) Neuroradiological features of contrast-induced neurotoxicity: A systematic review and pooled analysis. J Clin Neurosci 126:108–116 38870639 10.1016/j.jocn.2024.05.038

[13] Daneman R, Prat A (2015) The blood–brain barrier. Cold Spring Harb Perspect Biol 7(1):a020412 25561720 10.1101/cshperspect.a020412 PMC4292164

[14] Kadry H, Noorani B, Cucullo L (2020) A blood–brain barrier overview on structure, function, impairment, and biomarkers of integrity. Fluids and Barriers of the CNS 17:1–24 33208141 10.1186/s12987-020-00230-3 PMC7672931

[15] Mariajoseph FP, Lai L, Moore J, Chandra R, Goldschlager T, Praeger AJ et al (2024) Pathophysiology of contrast-induced neurotoxicity: a narrative review of possible mechanisms. Eur Neurol 87(1):26–35 38118425 10.1159/000535928 PMC11003557

[16] Bernardo-Castro S, Sousa JA, Brás A, Cecília C, Rodrigues B, Almendra L et al (2020) Pathophysiology of blood–brain barrier permeability throughout the different stages of ischemic stroke and its implication on hemorrhagic transformation and recovery. Front Neurol 11:1605 10.3389/fneur.2020.594672 PMC7756029 33362697

[17] Zevallos CB, Dai B, Dandapat S, Quispe-Orozco D, Holcombe A, Ansari S et al (2021) Greater intraprocedural systolic blood pressure and blood pressure variability are associated with contrast-induced neurotoxicity after neurointerventional procedures. J Neurol Sci 420:117209 33187680 10.1016/j.jns.2020.117209

[18] Zhang G, Li T (2018) The role of blood-brain barrier damage in the pathogenesis of contrast-induced encephalopathy. Arch Gen Intern Med 2(2):34–40

[19] Rapoport S, Thompson H, Bidinger JM (1974) Equi-osmolal opening of the blood-brain barrier in the rabbit by different contrast media. Acta Radiol Diagn 15(1):21–32 10.1177/028418517401500103 4821722

[20] Garcia-Polite F, Martorell J, Del Rey-Puech P, Melgar-Lesmes P, O’Brien CC, Roquer J et al (2017) Pulsatility and high shear stress deteriorate barrier phenotype in brain microvascular endothelium. J Cereb Blood Flow Metab 37(7):2614–2625 27702879 10.1177/0271678X16672482 PMC5531355

[21] Krueger M, Bechmann I, Immig K, Reichenbach A, Härtig W, Michalski D (2015) Blood—brain barrier breakdown involves four distinct stages of vascular damage in various models of experimental focal cerebral ischemia. J Cereb Blood Flow Metab 35(2):292–303 25425076 10.1038/jcbfm.2014.199 PMC4426746

[22] Bernardo-Castro S, Sousa JA, Brás A, Cecília C, Rodrigues B, Almendra L et al (2020) Pathophysiology of blood–brain barrier permeability throughout the different stages of ischemic stroke and its implication on hemorrhagic transformation and recovery. Front Neurol 11:594672 33362697 10.3389/fneur.2020.594672 PMC7756029

[23] Mariajoseph FP, Chung JX, Lai LT, Moore J, Goldschlager T, Chandra RV, Praeger A, Slater LA (2024) Clinical management of contrast-induced neurotoxicity: a systematic review. Acta Neurol Belg. 124(4):1141–9 38329641 10.1007/s13760-024-02474-4 PMC11266203

[24] Liu M-R, Jiang H, Li X-L, Yang P (2020) Case report and literature review on low-osmolar, non-ionic iodine-based contrast-induced encephalopathy. Clin Interv Aging 2277–89 10.2147/CIA.S280931 PMC7723034 33304098

[25] Dattani A, Au L, Tay KH, Davey P (2018) Contrast-induced encephalopathy following coronary angiography with no radiological features: a case report and literature review. Cardiology 139(3):197–201 29402812 10.1159/000486636

[26] Matsubara N, Izumi T, Miyachi S, Ota K, Wakabayashi T (2017) Contrast-induced encephalopathy following embolization of intracranial aneurysms in hemodialysis patients. Neurol Med Chir 57(12):641–648 10.2176/nmc.oa.2017-0132 PMC5735227 29093326

[27] Babalova L, Ruzinak R, Ballova J, Sivak S, Kantorova E, Kurca E et al (2021) Contrast-induced encephalopathy. Bratisl Lek Listy 122(9):618–620 34463105 10.4149/BLL_2021_098

[28] Donepudi B, Trottier S (2018) A seizure and hemiplegia following contrast exposure: understanding contrast-induced encephalopathy. Case Rep Med 2018(1):9278526 29686712 10.1155/2018/9278526 PMC5857315

[29] Şimşek EÇ, Ertürk E, Uçar R, Yilmaz AO, Ekmekçi C, Mutlu İ et al (2019) Transient contrast neurotoxicity after percutaneous coronary intervention mimicking subarachnoid hemorrhage in a patient with chronic kidney disease. Clinical Medicine Insights: Case Reports 12:1179547619867671 31413651 10.1177/1179547619867671 PMC6676254

[30] Health N, Council MR (2014) Ethical considerations in quality assurance and evaluation activities. NHMRC Canberra

