# Supplementary material for: The Australian Diagnostic Criteria for Contrast-Induced Encephalopathy
Source: Neuroradiology. 2025 Mar 29;67(5):1163–9. doi: 10.1007/s00234-025-03601-5 (PMC12125082; doi:10.1007/s00234-025-03601-5)
Supplement: Supplementary file 1 — Supplementary file1 (DOCX 13 KB) [file 234_2025_3601_MOESM1_ESM.docx]

Supplementary Table 1. Initial diagnostic items (presented in round 1)

| CIN should only be diagnosed if presentation is within 24 hours of contrast administration |
| --- |
| CIN should not be diagnosed if neurological signs can be explained by the presence of vessel occlusion or vascular territory ischaemia |
| Punctate diffusion restriction not correlating to symptoms does not necessitate vascular territory ischaemia |
| Perfusion abnormality without large vessel occlusion does not necessitate vascular territory ischaemia |
| CIN should not be diagnosed if neurological signs can be explained by intracranial haemorrhage |
| CIN should not be diagnosed if neurological signs s can be explained by a pre-existing seizure pattern in patients with known epilepsy |
| CIN should not be diagnosed if neurological signs can be explained by metabolic derangement. |
| CIN should not be diagnosed if neurological signs can be explained by the presence of intracranial malignancy |
| CIN should not be diagnosed if neurological signs can be explained by infection or delirium |
| CIN should not be diagnosed if neurological signs can be explained by recent head trauma |
| CIN is more likely if neurological symptoms are reversible |
| CIN is more likely if neuroimaging is normal |
| CIN is more likely in the presence of contrast staining that resolves on subsequent imaging |
| CIN is more likely in the presence of cerebral oedema resolves on subsequent imaging |
| CIN is more likely in the presence of cortical and subcortical signal MRI signal changes that resolve on subsequent imaging |
